# Toxicity of spinosad to temephos-resistant *Aedes aegypti* populations in Brazil

**DOI:** 10.1371/journal.pone.0173689

**Published:** 2017-03-16

**Authors:** Luciana dos Santos Dias, Maria de Lourdes da Graça Macoris, Maria Teresa Macoris Andrighetti, Vanessa Camargo Garbeloto Otrera, Adriana dos Santos Dias, Luiz Guilherme Soares da Rocha Bauzer, Cynara de Melo Rodovalho, Ademir Jesus Martins, José Bento Pereira Lima

**Affiliations:** 1 Laboratório de Fisiologia e Controle de Artrópodes Vetores, Instituto Oswaldo Cruz, Fiocruz, Rio de Janeiro, RJ—Brasil; 2 Laboratório de Entomologia, Instituto de Biologia do Exército, Rio de Janeiro, RJ—Brasil; 3 Superintendência de Controle de Endemias, Marília, SP—Brasil; Universidade Federal do Rio de Janeiro, BRAZIL

## Abstract

The mosquito *Aedes aegypti* is the primary vector of different arboviruses and represents a major public health problem. Several Brazilian populations of *Ae*. *aegypti* have developed resistance to temephos, the most used organophosphate larvicide. New tools which are less harmful to the environment and safer for humans are becoming increasingly important to control this insect vector. Spinosad, an aerobic fermentation product of a soil actinobacteria, has a favorable environmental profile. It presents selective insecticide properties, a mechanism of action that differs from those of many synthetic chemical insecticides. The toxicity of spinosad and temephos to *Aedes aegypti* populations from Brazil, which were previously exposed to temephos, were investigated in this study. Larval susceptibility (LC50) to temephos varied from 3μg/L for Rockefeller up to 260 μg/L for Santana do Ipanema field derived population. Larval susceptibility (LC50) to spinosad varied from 23μg/L for Rockefeller up to 93μg/L for Marilia field derived population. In addition, a semi-field trial was performed to evaluate spinosad (Natular^TM^ DT) initial efficacy and persistence toward four field-derived lineages and the Rockefeller lineage, used as an internal control. Spinosad was tested at 0.5mg active ingredient/L in 200L capacity water tanks. Mortality was recorded each 24 hours after exposition and tanks were further recolonized once per week with mortality being recorded daily for eight weeks. Spinosad provided a level equal or superior to 80% mortality during a seven to eight week evaluation period. The assessed populations did not present cross-resistance between spinosad and temephos in laboratory conditions. It demonstrates that spinosad may be a promising larvicide for the control of *Ae*. *aegypti*, especially for populations in which resistance to temephos has been detected.

## Introduction

The *Aedes aegypti* (Linnaeus, 1762) mosquito, widely distributed in the tropical and subtropical regions of the planet, is highly adapted to the urban environment and is often found within and around households [1–3]. It is the main vector of dengue viruses and can play a role as vector of yellow fever virus in the urban cycle [4], which are considered two of the most important viral diseases transmitted by arthropods [5]. Additionally, this insect is a potential vector transmitter of Chikungunya virus and Zika virus [6–8]. Efficient vaccines against dengue, chikungunya and zika are not currently available. Because of that, public health measures focus mainly on the vector control [9,10].

Control programs aim to reduce the populations of insects that transmit ethiological agents by eliminating potential breeding sites or by using insecticides [11–13]. In Brazil, the control of dengue vector larvae has been carried out for decades with the use of temephos, an organophosphate larvicide. Resistance to this compound has been reported since 1998 [14–16]. Nowadays the majority of the evaluated Brazilian populations is resistant to temephos [14,17–20].

Insecticide resistance is the heritable ability of the insect to survive to insecticide concentrations that are lethal to individuals of the same species (susceptible ones) [21]. The major mechanisms recorded for *Ae*. *aegypti* resistance to temephos has been associated with changes at its target site in the acetylcholinesterase. It has also been associated with metabolic mechanisms related with enzymes involved in the detoxification of xenobiotic compounds, named metabolic resistance [22–24]. The insecticide metabolic resistance can involve three major enzyme groups: esterases (ESTs), glutathione-S-transferases (GSTs) and mixed function oxidases (MFOs) [25]. Detoxifying enzymes can confer resistance to many chemicals belonging to the same group or to different groups, characterizing the cross resistance [26].

In this sense, alternatives to organophosphates for the control of *Ae*. *aegypti* and other insects of medical importance are essential [27,28]. Also, there are reports of *Ae*. *aegypti* resistance to the main insecticides in use [18]. Alternatives to organophosphates, such as, the insect growth regulators (IGRs), *Bacillus thuringiensis* serovar *israelensis* (*Bti*) and spinosad, which are environmental safe and innocuous for humans, have attracted growing interest. Spinosad is composed of two natural metabolites, spinosyns A and D [29], which are products of the aerobic fermentation of *Sacharopolyspora spinosa*, a soil actinobacteria [30,31]. Spinosad acts on both the nicotinic acetylcholine receptors and the ү-aminobutyric acid (GABA) receptors, causing excitation of the insect’s nervous system, paralysis and death [32–34]. Therefore, although also neurotoxic, spinosad has a distinct molecular target when compared to organophosphates and pyrethroids, which target the enzyme acetilcholynesterase and the voltage gated sodium channel, respectively [23].

Tests of chronical toxicity demonstrate that spinosad does not present carcinogenic, teratogenic or neurotoxic effects in mammals [35], and has a favorable profile with low environmental persistence. It displays low toxicity for non-targeted insects and it is innocuous for fish and other aquatic vertebrates, when used in the recommended dosages [36–38]. Marcombe et al. [39] noticed that spinosad is a promising larvicide against *Ae*. *aegypti* on water reservoirs, where its residual efficacy lasted for up to 16 weeks. Moreover spinosad also showed high toxicity against other mosquitoes species as *Anopheles stephensi*, *Anopheles albimanus* and *Culex quinquefasciatus* [40–43]. Recently the World Health Organization Pesticide Scheme (WHOPES) approved spinosad for usage in drinking water [43]. However, possible cross-resistance selected by other compounds needs to be investigated before its wide utilization.

There is a high level of resistance to organophosphates and pyrethroids in worldwide *Ae*. *aegypti* populations. Moreover, spinosad and temephos have action on different target sites, which supports the lack of cross-resistance, considering this mechanism. However, we can not discard the hypothesis that cross-resistance could be found due to metabolic detoxification mechanism selected by temephos. Because of that it is important to evaluate the efficacy of spinosad over populations with distinct profiles of resistance to temephos. In this study, we assessed and compared the toxicity of spinosad and temephos insecticide in Brazilian *Ae*. *aegypti* populations with putative different genetic backgrounds, under laboratory and field (simulation) conditions.

## Materials and methods

**Fig 1:**
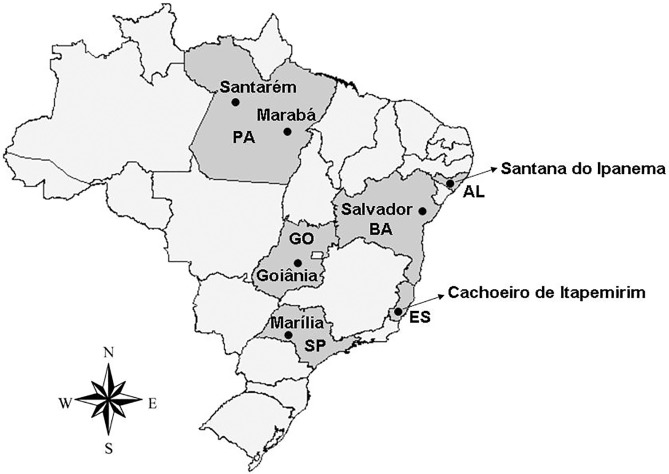
Brazilian political map showing the states where collections were performed.

1) Mosquitoes–Seven *Ae*. *aegypti* populations were collected in six different Brazilian states, representing four geopolitical regions of the country: Santarém and Marabá (State of Pará, Northern Brazil), Goiânia (State of Goiás, Midwestern Brazil), Cachoeiro de Itapemirim and Marília (State of Espírito Santo and State of São Paulo, respectively; Southeastern Brazil), Salvador and Santana do Ipanema (State of Bahia and State of Alagoas, respectively; Northeastern Brazil) (Fig 1). The eggs were collected in the field with the help of ovitraps containing hay infusion [44], which was distributed in a way to represent the whole geographical extension of the localities mentioned above [15]. These materials were collected by the municipalities' health secretaries and sent to the Laboratório de Fisiologia e Controle de Artrópodes Vetores (Fiocruz, Rio de Janeiro) by the Brazilian Health Ministry.The females were reared as follow: eggs collected in the field were hatched in the laboratory in order to obtain 1^st^ instar larvae that developed to the adults of the parental generation of these strains. Approximately 1000 larvae were reared in plastic bowls containing 1L of dechlorinated water plus one gram of cat food (Friskies, Purina). The food was provided every three days until the larvae reached the pupae phase. The pupae were separated in plastic cups of 50ml and put inside cardboard cages until adults emergence. Adults were kept in a controlled environment with temperature of 26°C ± 1 and 70% humidity, and fed *ad libitum* with a solution of 10% sucrose. Guinea pigs *Cavia porcellus* (Linnaeus, 1758; Rodentia, Caviidae) were used for blood feeding, with approval of the Ethics Committee for Animal Use from Fundação Oswaldo Cruz (CEUA—Fiocruz) n° L -011/09.

2) Insecticides–Temephos (PESTANAL^®^ - 95,6% temephos a.i., Sigma–Aldrich) and liquid spinosad (Natular^TM^ 20EC– 20.6% Spinosad a.i., Clarke Mosquito Control Products, Inc.) were used for laboratory assays. The insecticides were diluted so that a final concentration of 3000mg/L was achieved for both. After normalizing the concentration, we prepared the solutions for the bioassays. For the field simulations, one effervescent tablet of spinosad (Natular^TM^ DT– 7.48% Spinosad a.i., Clarke Mosquito Control Products, Inc.) was used per 200 L tank, which provided a final concentration of 0.5 mg active ingredient/L, as recommended by WHO [45].

3) Laboratory tests–Assays were performed in the Laboratório de Fisiologia e Controle de Artrópodes Vetores, Instituto Oswaldo Cruz–Fiocruz, located at the Instituto de Biologia do Exército (IBEx), Rio de Janeiro, Brazil. The F1 and F2 generations descending from the seven field populations were used. The Rockefeller lineage, which is a reference of susceptibility to insecticides [46], was used as control for each experiment. This lineage was reared in the same conditions as described for the field colonies. The larvae used in the laboratory bioassays were reared as follow: 1,000 larvae from each population were kept in plastic bowls containing 1L of water and one gram of cat food (Friskies, Purina) until they reached L3 instar for bioassay performances.

Bioassay with temephos was conducted based on the World Health Organization (WHO) protocol [47]. For each dosage, 80 L3 larvae (four replicas containing 20 larvae each in white plastic cups containing 100ml of solution) were exposed to a minimum of 10 to 12 different doses of insecticide (between 1.05 and 720μg/L). The assays were conducted four times, on different days, for each of the seven populations and the Rockefeller lineage. The temephos solution was prepared with ethanol and dechlorinated water. For the control group, only ethanol (0.6%) was used. Mortality reading was performed after 24h of exposure.

Before the spinosad dose-response bioassays, we first evaluated the time of exposure to the product (24h, 48h or 72h). Third instar Rockefeller larvae were exposed to 10 spinosad concentrations, ranging from 10 to 70μg/L. For each concentration, four replicas were tested using 10 L3 larvae each, in transparent plastic cups containing 150mL of solution. The spinosad solution was prepared with ethanol and dechlorinated water. For the control group, only ethanol (0.4%) was used. Mortality was recorded at 24, 48 and 72 hours of exposure. The same experimental set was analyzed at different periods. 14mg of triturated cat meal (Friskies; Purina) per plastic cup was provided in the first day of the bioassays. The assays were conducted three times, on different days. Dose-response bioassays with natural populations were performed with 10 to 12 spinosad concentrations (between 10 and 300μg/L), as described above, with 24 hours of exposure.

4) Semi-field simulation assays–The experiments were conducted at the external area of SUCEN—Superintendência de Controle de Endemias—Laboratório de Entomologia Aplicada in Marília city, São Paulo State. The city climate is rainy tropical with a dry winter. The temperature ranges from 17 to 29°C [48].

Once the laboratory experiments were concluded, four natural populations were still available for carrying out field tests simulations. Those samples were sent to SUCEN, in Marilia city, by the Laboratório de Fisiologia e Controle de Artrópodes Vetores (Fiocruz, Rio de Janeiro). The populations were divided in two groups, the first comprised Marília (F1 generation) and Salvador (F1 generation) populations which were tested from July 2011 to February 2012. The second group comprised Goiânia (F2 generation) and Santarém (F1 generation) populations, which were tested from May to November 2012. In both cases, Rockefeller reference population was used as a control.

Tests were performed outside the laboratory in a covered area to protect the tanks from environmental factors such as rain. Eighteen polyethylene water tanks filled with 200L of tap water were used to conduct the tests. In order to simulate residential usage situation, a third of the water volume of the tank was replaced three times a week.

For each test, 12 tanks were treated (E) and six remained untreated (C) as control. In order to avoid bias in the results related to tank position, larvae from a single population were tested in all tanks, placed in alternated positions, passing for each tank at least once. The day of the treatment was considered the "day zero", and 30 3rd instar larvae were used to colonize each tank. The larvae were previously reared in plastic bowls containing 1L of water and one gram of cat food (Friskies, Purina). The larvae were placed into each water tanks. Soon after, one tablet of the Natular DT was added at the center of the ‘E’ tanks, as recommended by the manufacturer. Every week, surviving larvae were removed and a new batch of 30 3rd instar larvae was added to each tank. Food (Friskies; Purina) was provided for larvae in the first day and each three days of the experiment, if necessary.

The mortality was recorded 24 h after the exposure and then daily, at the same time. The water temperature and water pH of the tanks were monitored daily with the assistance of a thermo-hygrometer (Fisher Scientific^®^) and a Ph Meter (Orion Research Inc., Cambridge, MA).

The larvicide effect was evaluated for an eight-weeks period, which equals to the residences visiting cycle intervals of the National Dengue Control Program. Each test was repeated three times on different periods, thus completing one evaluation. The two first tests of one evaluation were conducted with covered water tanks, while in the third test the tanks were covered with nylon mesh to prevent entry of insects and eventual detritus. By using different covers, we wanted to evaluate eventual environmental influences over the residual effect of the product, such as the influence of indirect solar radiation in the tanks.

5) Statistical analysis—To estimate the lethal concentrations (LCs) for each type of treatment and their respective confidence interval, probit analysis was conducted using the Polo-PC software [49]. The resistance ratios (RR) were obtained through the division of LCs 50 and 95 of the field populations by the respective LCs of the Rockefeller lineage. The comparison of lethal concentration (LC_95_) between the different times of exposure to spinosad was conducted through the overlap of its 95% confidence intervals. A linear regression analysis was performed to check if there was a correlation between the RR95 of the analysed populations to temephos and spinosad. The above mentioned analysis were all performed with the help of the Graph-Pad Prism software version 5.0 for Windows (GraphPad Software, San Diego, California, USA). A test t followed by Mann-Whitney test was performed to compare the effectiveness of spinosad in water tanks covered with a lid or with nylon mesh, with the help of the Graph-Pad Prism software version 5.0 for Windows (GraphPad Software, San Diego, California, USA).

## Ethics statements

No specific permissions were required as the mosquito collections were performed by the municipalities’ health secretaries as requested by the Brazilian Health Ministry. The species used in this study (*Aedes aegypti*) is not an endangered or protected species. Mosquito blood-feeding on anesthetized guinea pigs (Cavia porcellus) was authorized by Fiocruz Ethical Committee (CEUA 011/09).

## Results

### Larval susceptibility

The comparisons of the LCs95 averages were performed by superimposing the 95% confidence intervals of lethal concentrations at each evaluated exposure time (S1 Table). An overlap occurred between 24, 48 and 72h exposure indicating no significant difference. Therefore, it was determined that the mortality reading could be done after 24 hours exposure of *Ae*. *aegypti* to spinosad.

**Fig 2:**
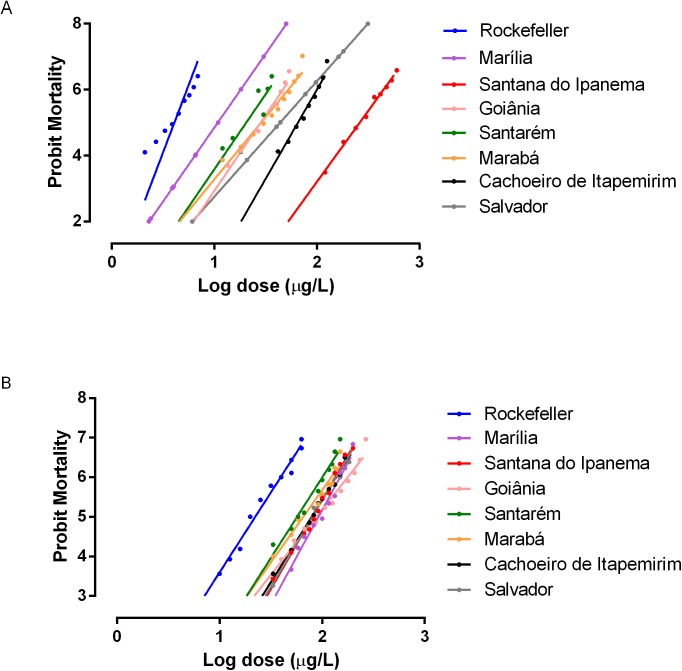
**Mortality of *Ae*. *aegypti* larvae exposed for 24h to temephos (A) and spinosad (B) insecticides**.

The comparative analysis of the spinosad and temephos LCs toward the reference Rockefeller colony has shown that the latter product required a smaller dose to kill 50% of the larvae (Table 1). The lethal concentration (LC50) of the larvae bioassays for spinosad ranged from 23μg/L for the susceptilble Rockefeller reference lineage up to 82μg/L for the field populations. For themephos the lethal concentration of the bioassays with larvae ranged from 3μg/L for Rockefeller up to 260μg/L for field populations. Although the LC50 for temephos was lower for the Rockefeller lineage, there was great variation in the LC50 for the field populations exposed to the product. For spinosad the LC50 in the Rockefeller lineage was higher but the variation in LC50 among the field populations was narrower. The field populations used in this study were tested to verify the susceptibility/resistance status to the larvicide organophosphate temephos and eventual cross-resistance to the spinosad. The amplitude of the dose-response effect to both insecticides can be observed in Fig 2. For organophosphates a classification of populations resistance can be build using the criteria proposed by Mazzarri and Georghiou [50]. In this criteria a RR (resistance ratio) lower than 5 indicates low resistance, RR between 5 and 10 indicates intermediate resistance and over 10 indicates a high resistance.The levels of spinosad toxicity were similar for all evaluated populations. RR_50_ ranged from 2.5 (Santarem) to 4.1 (Marilia) and RR_95_ ranged from 2.8 (Santarém) to 5.4 (Goiânia). Different levels of resistance were observed to temephos, with RR_50_ ranging from 2.3 (Marília) to 89.8 (Santana do Ipanema) and RR95 ranging from 3.6 (Marília) to 119.1 (Santana do Ipanema), as seen in Table 1. No significant correlation between the RR_95_ for temephos and spinosad was observed (R^2^ = 0.0368; p = 0.6803).

**Table 1:** Toxicity of temephos and spinosad against 3rd instar *Aedes aegypti* larvae after a 24h exposure.

**Temephos**								
**Population**	**Number of larvae**	**Slope**	**LC50 (er of**	**CI 95%**	**RR50**	**LC95 (5% of**	**CI 95%**	**RR95**
Rockefeller	**2400**	**6.2**	**3**	**2.80–2.98**	**1**	**4.7**	**4.54–4.78**	**1**
Santarém / PA	2640	4.3	19	18.24–19.37	6.5	45	42.93–48.49	8.5
Marabá / PA	2640	3.8	29	25.62–31.39	9.9	78	66.28–91.31	14.6
Goiânia / GO	2640	4.5	28	25.83–30.90	9.8	65	55.5–75.73	12.2
Cachoeiro de Itapemirim / ES	2640	5.6	65	61.86–69.33	22.7	129	118.68–140.97	24.3
Marília / SP	2400	4.5	6.9	6.60–7.30	2.3	17	15.00–18.00	3.6
Salvador / BA	2640	4.5	33	25.00–58.00	11.0	78.5	71.00–92.00	16.7
Santana do Ipanema / AL	2880	4.2	260	251.25–267.74	89.8	635	602.09–673.82	119.1
**Spinosad**								
**Population**	**Number of larvae**	**Slope**	**LC50 (er of**	**CI 95%**	**RR50**	**LC95 (5% of**	**CI 95%**	**RR95**
Rockefeller	1200	**4.3**	**23**	**21.63–23.80**	**1**	**55**	**50.96–60.01**	**1**
Santarém / PA	1440	3.9	57	53.89–60.66	2.5	152	139.62–168.65	2.8
Marabá / PA	1320	3.5	66	61.71–69.92	2.9	193	174.06–219.96	3.5
Goiânia / GO	1440	5.3	89	83.61–94.46	3.9	299	267.67–340.33	5.4
Cachoeiro Itapemirim / ES	1440	5.2	80	76.72–83.20	3.5	198	182.94–218.66	3.6
Marília / SP	1200	5.1	93	89.02–96.82	4.1	214	196.98–235.63	3.9
Salvador / BA	1440	4.2	80	76.15–83.96	3.5	195	181.42–213.27	3.6
Santana do Ipanema / AL	1440	4.6	82	78.63–85.17	3.6	187	173.58–203.52	3.4

RR = Resistance ratio LC = Lethal concentration, CI 95% = confidence interval 95% Resistance Ratio (RR) RR<5 = low resistance, RR between 5 and 10 = intermediate resistance, RR>10 = high resistance.

### Evaluation of spinosad persistence under semi-field conditions

**Fig 3:**
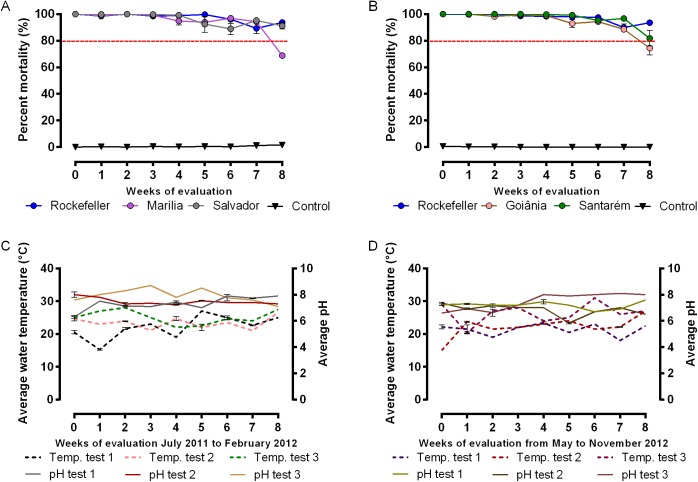
Field evaluation of Spinosad (Natular^TM^ DT) against *Aedes aegypti* 3rd instar larvae. The average mortality percentage is represented for three independent bioassays conducted in a row, A- Rockefeller, Marilia and Salvador (tested between July 2011 and February 2012). B- Rockefeller, Goiânia and Santarém (tested between September 2011 and November 2012). C- pH and temperature of the solution in the tanks during the three assays for the Rockefeller, Marilia and Salvador group. D- pH and temperature of the solution in the tanks during the three assays for the Rockefeller, Goiânia and Santarém group. The dose tested was 0.5mg/L. Every week the tanks were recolonized by removing the dead and the remaining living larvae and introducing 30 new 3rd instar larvae.

The data related to the residual effect of spinosad (Natular DT) in simulated field are shown in Fig 3. Spinosad treatment showed persistence during a seven to eight-week period providing at least 80% of larvae mortality for two populations, Marilia (Fig 3A) and Goiânia (Fig 3B). For the remaining populations there was no decrease up to the eighth week. There was a small loss of the control population in one of the triplicates (S1C Fig) probably due to the high temperatures that occur in Marilia city in February.

The tanks were covered either with a lid (1^st^ and 2^nd^ replicates) or with a nylon mesh (3^rd^ replicate). Regardless how the water tanks were covered, the mortality rate was not significantly different for each population, as revealed by the Mann Whitney tests (P>0.05 for all comparisons). The average water temperature was 23.5°C ± 3.1 and pH of the test was 7.4 ± 0.6 (Fig 3C and 3D). The details of the tests for each population are shown in S1 Fig and S2 Fig.

The mortality effect of spinosad tended to be constant over the weeks, with reduction in mortality starting in the seventh week for the majority of populations. When the experiments were analyzed individually, small variations were observed. For example, there was as decline in mortality percentage (<80%) in the population of Salvador after the sixth week, increasing to rates above 80% in the following week (S1A Fig). In the third test (S2C Fig), Goiania mortality rate was 72% in the seventh week.

## Discussion

The *Ae*. *aegypti* resistance to chemical products has been reported around the globe [50–56]. In Brazil, the detection of populations resistant to the temephos larvicide started in 1998 [14–16]. Because of this, additional to elimination or correct treatment of larvae breeding sites, there was a need to search for alternative compounds for *Ae*. *aegypti* control. Spinosad, with a low toxicity for mammals and the environment, and being neurotoxic for *Ae*. *aegypti*,seems to be a good option. However, there is a possibility it can be detoxified by cross-resistance. Despite of this, it may yet be an important tool to combat vectors already resistant to other products.

According to WHO, studies to estimate the lethal concentration for *Ae*. *aegypti* larvae in the laboratory must be conducted using reference lineages. In addition, the larvae must remain exposed for over 24 hours to the larvicide [57]. Many authors have conducted studies of spinosad effect on *Ae*. *aegypti* larvae from laboratory colonies, however, the protocol and the time of exposure varied in each work [29,41,58–60]. In this study, procedures for experiment standardization using spinosad dose response curve were conducted with the Rockefeller lineage (L3 larvae) and mortality was scored after 24, 48 and 72 hours of exposure to the insecticide. This was performed as a preliminary step to confirm the correct period for mortality recording. Comparing the LC95 for each period, we noticed that there was an overlap in their interval of confidence indicating that the differences found were not significant, therefore leading us to keep 24 hours of exposure as ideal for dose-dependent bioassays with spinosad.

Studies performed with spinosad showed great variation on the lethal concentrations obtained using laboratory populations of *Ae*. *aegypti*: Darriet et al [29], Romi et al [58] and Paul et al [59] found a LC50 of 35μg/L, 10μg/L and 160μg/L respectively. However, Bond et al [41] and Pérez et al [60] used the same protocol and obtained similar LC50, 25μg/L and 26μg/L, respectively. These results are similar to the results we obtained in dose-response curve of spinosad over the susceptible Rockefeller lineage (LC50 = 23μg/L). It indicates that spinosad displayed high toxicity to *Ae*. *aegypti* reference colony tested and its lethal concentration is very low. However, the lethal concentration for the reference colony was not as low as that found for temephos (LC50 = 3.0μg/L). Here it is worth remembering that the temephos and spinosad used to determine toxicity in the biossays have differences in their active ingredient contents, which are 95.6 and 20.6%, respectively. The discrepancy found by other authors may be attributed to differences in protocols used in each laboratory, which highlights the need to maintain a standard protocol that facilitates the comparison of results from different groups.

The field populations assessed in this study were characterized as resistant to the temephos organophosphate based on the criteria proposed by Mazzarri and Georghiou [50]. However, the RRs for spinosad in all populations were extremely low when compared to the RRs obtained for temephos. Only the population of Goiânia presented RR>5 (RR at LC95 = 5.4) for spinosad. Since none of these populations have been treated with spinosad before, all the RRs observed seem to be variations of the populations susceptibility rather than resistance to spinosad. Interestingly, Goiânia population was not the one with the highest RR95 to the organophosphate temephos. The population of Santana do Ipanema showed the highest RR95 for temephos and a very low RR95 for spinosad. As expected, no significant correlation was noted between the RR95 of the populations to temephos and spinosad (R2 = 0.0368; p = 0.6803), suggesting that the resistance mechanisms selected to temephos are not influencing the toxicity levels of spinosad. Some studies suggested that once spinosad has different targets in the insect’s nervous system, there is a lower possibility of cross-resistance with other products used in the control of disease vectors [31,33,34], corroborating our results.

During the assessment of spinosad residual effect on semi-field simulation, the average temperature recorded in the recipients was 23.5 ± 3.1°C, and the average of the pH was 7.4 ± 0.6. Other semi-field simulation study has shown that *Bti* larvicides efficiency had an inverse relationship between temperature (which ranged from 18.7 to 34.6°C during the simulation) and the persistence of the *Bti* on *Ae*. *aegypti* larvae [61]. This may be a problem, since the dengue vector proliferates in tropical and subtropical areas [62]. Fontoura et al [16], verified that the climatic variation, even in the period when elevated temperatures were not recorded on a field simulation, influenced negatively on the persistence of IGR Novaluron, with an increase in emergency of adults a week earlier if compared to simulations conducted in the periods when the temperature remained stable. In our results, it was not possible to verify this influence, probably due to the low temperature variation. In addition, we also did not observe the interference of changes in the water pH, maybe because the variation during the assessments was very small. These alterations were not relevant, even in the recipients of the third assessment, which instead of a solid cover had a nylon mesh attached with a rubber band to seal the water tank. As observed in the mortality graphs, our results suggest that a higher exposure of the tank’s content to the environmental variations, and a faster evaporation than that observed on the sealed recipients did not influence, in a significant manner, the residual effect of the product. A possible influence of the indirect insolation could potentially be detected since spinosad is vulnerable to photolysis [63]. Pérez et al. [60] verified that the half life of aqueous solutions of spinosad on *Ae*. *aegypti* larvae in plastic containers containing 1L of solution (10mg/L) differed according to the exposition of direct sun light or not. When exposed to sunlight conditions the half-life was 2.1 days. When allocated in the shadow the half life was 24.5 days. However, in our studies, since no statistic difference was observed between the two forms of treatment, the indirect insolation, if it occurred, did not have any critical effect.

A similar experiment conducted by Thavara et al. [64], showed that Spinosad DT, at 0.5mg/L in clay jugs, with 200L of tap water and weekly colonized with 25 third instar *Ae*. *aegypti* larvae, achieved a persistence period of 27 days. In our assessments with the same spinosad concentration, there was a minimum persistence of eight weeks (56 days) in every population (mortality higher than 80%). In other semi-field evaluation using plastic bowls also containing 1L of of spinosad aqueous solution (1mg/L) the persistence was kept for eight weeks on *Ae*. *aegypti* larvae [41]. Differences in the nature of the recipients used on the field simulations may justify divergences on results.

In Brazil, the visit of health agents on households to check and treat possible foci happens every eight weeks [65]. Spinosad appeared to be efficient against Brazilian *Ae*. *aegypti* populations, presenting larvae mortality over 80% for seven to eight weeks. Therefore, schedule of mosquito control measures carried out by the health agents can be maintained. New assessments on a field simulation with reservoirs of different natures, expositions to different light and temperature conditions will be important to measure the product’s effect on various environments.

## Conclusion

The data presented here suggests that there is no cross-resistance of the spinosad with the temephos, regardless the resistance level to the organophosphate. Spinosad is a less aggressive product towards the environment and presents low toxicity for non-targeted insects and low risk for humans and fauna. Also, it has long residual effect on field populations with different levels of resistance. Therefore, spinosad is showing to be a promising product for public health usage, especially if used carefully. However, deeper studies that better evaluate the action mechanisms of spinosad in Brazilian *Ae*. *aegypti* populations are necessary to foresee possible events of tolerance and resistance to the product and to avoid troubles arising from the inadequate use of this tool.

## Supporting information

S1 Table Lethal concentration average and Confidence Interval (CI) according to exposure time of Rockefeller larvae to spinosad. (DOCX)

S1 Fig Field simulation for evaluation of the spinosad effect over the mortality of *Ae*. *aegypti* larvae. The average of mortality percentage for Rockefeller, Marilia and Salvador is represented for bioassays conducted in three distinct moments:
A–July to September 2011, B–September to November 2011 and C- December 2011 to February 2012. The red dotted line indicates the mortality level at 80%. The physicochemical variations, pH and temperature of the solution in the deposits during the three assays are represented in D. The dose tested was 0.5mg/L. Every week the tanks were recolonized by removing the dead and the remaining living larvae and introducing 30 new 3rd instar larvae. The tanks were covered either with a lid (1^st^ and 2^nd^ replicates) or with a nylon mesh (3^rd^ replicate).
(TIF)

S2 Fig Field simulation for evaluation of the spinosad effect over the mortality of *Ae*. *aegypti* larvae. The average mortality percentage for Rockefeller, Goiânia and Santarém is represented for bioassays conducted in three distinct moments: A- May to June 2012, B–July to September 2012 and C–September to November 2012. The dotted red line indicates the mortality level at 80%. The physicochemical variations, pH and temperature of the solution in the deposits during the three assays are represented in D. The dose tested was 0.5mg/L. Every week the tanks were recolonized by removing the dead and the remaining living larvae and introducing 30 new 3rd instar larvae. The tanks were covered either with a lid (1^st^ and 2^nd^ replicates) or with a nylon mesh (3^rd^ replicate).
(TIF)
