# Supplementary material for: Toxicity of spinosad to temephos-resistant Aedes aegypti populations in Brazil
Source: PLoS One. 2017 Mar 16;12(3):e0173689. doi: 10.1371/journal.pone.0173689 (PMC5354417; doi:10.1371/journal.pone.0173689)
Supplement: S1 Table — (DOCX) [file pone.0173689.s001.docx]

S1 Table

|  | LC95%* (Average) | CI** (min) | CI (max) |
| --- | --- | --- | --- |
| Rockefeller 24 hours | 0,05496 | 0,05096 | 0,06001 |
| Rockefeller 48 hours | 0,04594 | 0,04165 | 0,05189 |
| Rockefeller 72 hours | 0,04026 | 0,03676 | 0,04496 |

*LC95%=Lethal Concentration 95% **CI=Confidence Interval
