# Supplementary figures and images for: Toxicity of spinosad to temephos-resistant Aedes aegypti populations in Brazil
Source: PLoS One. 2017 Mar 16;12(3):e0173689. doi: 10.1371/journal.pone.0173689 (PMC5354417; doi:10.1371/journal.pone.0173689)

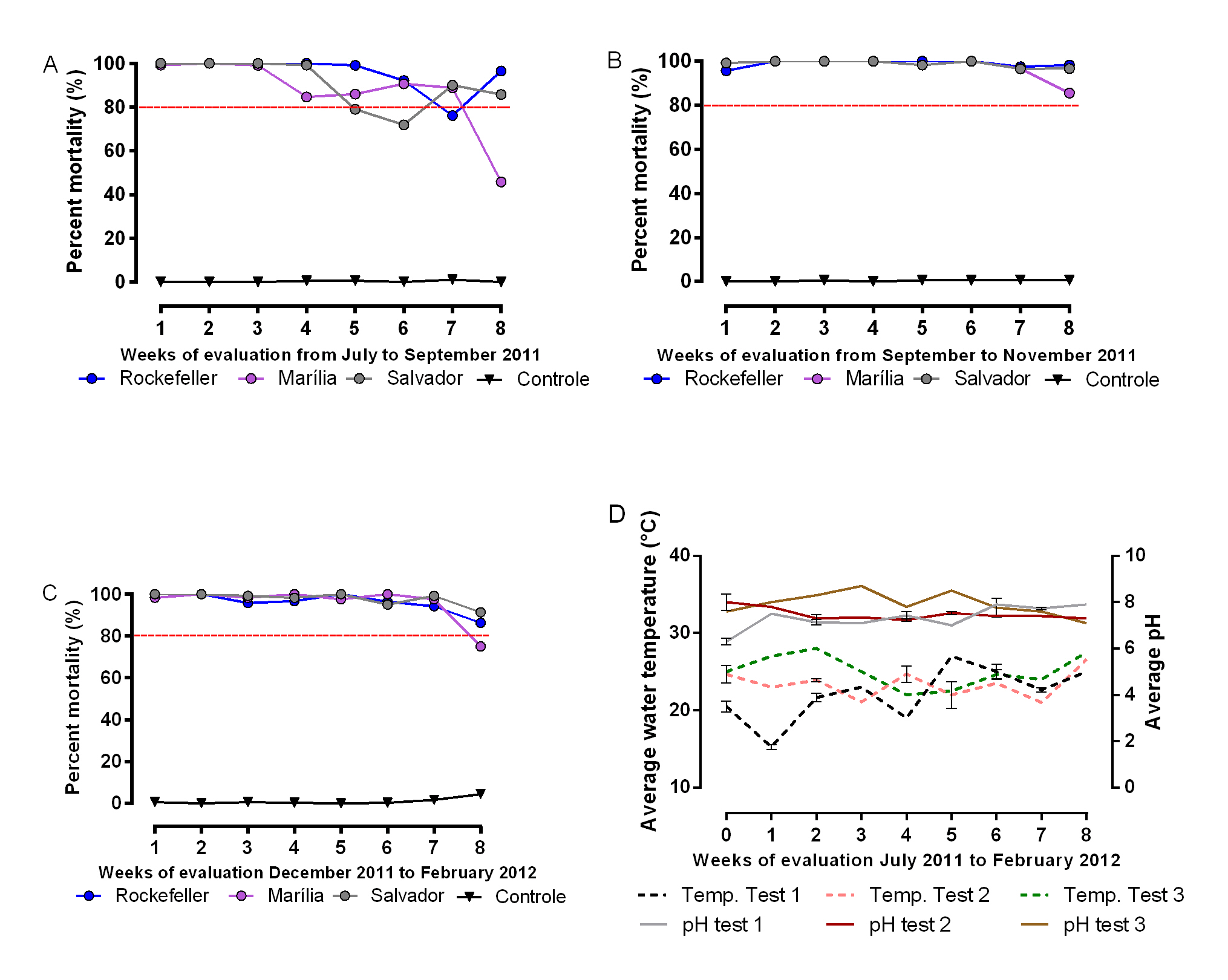

Supplement: S1 Fig — The average of mortality percentage for Rockefeller, Marilia and Salvador is represented for bioassays conducted in three distinct moments: A–July to September 2011, B–September to November 2011 and C- December 2011 to February 2012. The red dotted line indicates the mortality level at 80%. The physicochemical variations, pH and temperature of the solution in the deposits during the three assays are represented in D. The dose tested was 0.5mg/L. Every week the tanks were recolonized by removing the dead and the remaining living larvae and introducing 30 new 3rd instar larvae. The tanks were covered either with a lid (1st and 2nd replicates) or with a nylon mesh (3rd replicate). (TIF) [file pone.0173689.s002.tif]

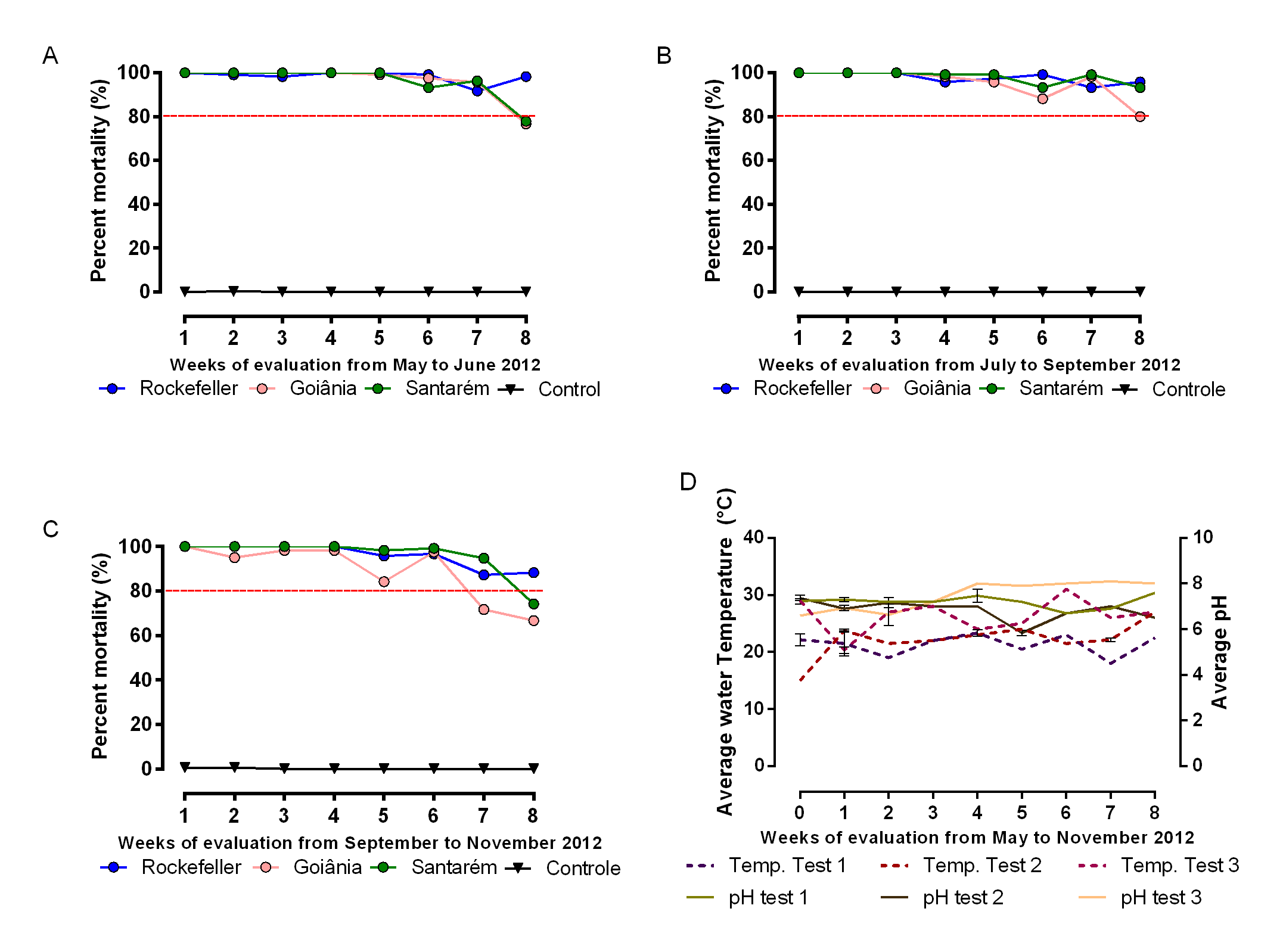

Supplement: S2 Fig — The average mortality percentage for Rockefeller, Goiânia and Santarém is represented for bioassays conducted in three distinct moments: A- May to June 2012, B–July to September 2012 and C–September to November 2012. The dotted red line indicates the mortality level at 80%. The physicochemical variations, pH and temperature of the solution in the deposits during the three assays are represented in D. The dose tested was 0.5mg/L. Every week the tanks were recolonized by removing the dead and the remaining living larvae and introducing 30 new 3rd instar larvae. The tanks were covered either with a lid (1st and 2nd replicates) or with a nylon mesh (3rd replicate). (TIF) [file pone.0173689.s003.tif]
